# Overexpression of *PeHKT1;1* Improves Salt Tolerance in *Populus*

**DOI:** 10.3390/genes9100475

**Published:** 2018-09-29

**Authors:** Meng Xu, Caihui Chen, Heng Cai, Ling Wu

**Affiliations:** 1 Co-Innovation Center for Sustainable Forestry in Southern China, Nanjing Forestry University, No. 159 Longpan Road, Nanjing 210037, China; chencaihui@njfu.edu.cn (C.C.); hengcai@njfu.edu.cn (H.C.); doriosea@sina.com (L.W.); 2 College of Forestry, Nanjing Forestry University, No. 159 Longpan Road, Nanjing 210037, China

**Keywords:** *PeHKT1;1*, salt tolerance, overexpression, *Populus*

## Abstract

Soil salinization is an increasingly serious threat that limits plant growth and development. Class I transporters of the high-affinity K^+^ transporter (HKT) family have been demonstrated to be involved in salt tolerance by contributing to Na^+^ exclusion from roots and shoots. Here, we isolated the *PeHKT1;1* gene from hybrid poplar based on the sequences of the *Populus trichocarpa* genome. The full-length *PeHKT1;1* gene was 2173 bp, including a 1608 bp open reading frame (ORF) encoding 535 amino acids and containing eight distinct transmembrane domains. Multiple sequence alignment and phylogenetic analysis suggested that the PeHKT1;1 protein had a typical S–G–G–G signature for the P-loop domains and belonged to class I of HKT transporters. *PeHKT1;1* transcripts were mainly detected in stem and root, and were remarkably induced by salt stress treatment. In further characterization of its functions, overexpression of *PeHKT1;1* in *Populus davidiana* × *Populus bolleana* resulted in a better relative growth rate in phenotypic analysis, including root and plant height, and exhibited higher catalase (CAT), peroxidase (POD), and superoxide dismutase (SOD) activities than non-transgenic poplar under salt stress conditions. These observations indicated that *PeHKT1;1* may enhance salt tolerance by improving the efficiency of antioxidant systems. Together, these data suggest that *PeHKT1;1* plays an important role in response to salt stress in *Populus*.

## 1. Introduction

In the natural environment, soil salinization is a major abiotic stress that limits plant growth and development. High concentrations of salts in the soil have various adverse effects in plants, including osmotic stress and ion toxicity. Specifically, excess salinity decreases water potential in plants, resulting in a reduced ability to take up water, and large amounts of sodium (Na^+^) and chloride (Cl^−^) are taken up by the plant root system. Excessive Na^+^ and Cl^−^ within plants is toxic, and disturbs potassium (K^+^) homeostasis, cellular activity, metabolism, and photosynthesis, and causes the accumulation of reactive oxygen species (ROS) [1,2,3]. To cope with soil salinization, plants have evolved diverse adaptive mechanisms, including Na^+^ exclusion from the shoot, Na^+^ expulsion from cell cytoplasm, and Na^+^ compartmentalization into vacuoles [4]. Among these, Na^+^ extrusion out of the cell and detoxification into vacuoles have been reported to be mediated by Salt-Overly-Sensitive 1 (SOS1) antiporters and Na^+^/H^+^ exchanger 1 (NHX1) antiporters, respectively [4,5]. The regulation of Na^+^ loading into the root xylem, which is limiting to Na^+^ accumulation in the shoot, is essential for enhancing salt tolerance. Therefore, it is necessary to understand target genes for Na^+^ extrusion from the shoot.

The high-affinity K^+^ transporters (HKTs) are a large superfamily of transporters in plants, bacteria, and fungi. *TaHKT2;1* was first identified in plants from bread wheat (*Triticum aestivum*) and encoded a Na^+^–K^+^ co-transporter [6,7]. Subsequently, more HKT transporters from other plant species were discovered in regard to salinity tolerance, including *Arabidopsis thaliana*, rice (*Oryza sativa*), barley (*Hordeum vulgare*), soybean (*Glycine max*), *Eucalyptus camaldulensis*, *Suaeda salsa*, *Salicornia europaea*, *Puccinellia tenuiflora*, and *Fragaria* spp. [8,9,10]. According to their structure and transport properties, HKT transporters are classified into at least two subfamilies [11]. Class I HKT transporters, reported in monocotyledonous and dicotyledonous species, are low-affinity transporters with specificity for Na^+^ [3]. As a typical HKT1 transporter, *AtHKT1;1* from *Arabidopsis* clearly shows the role of HKT1 transporters in plant salt tolerance, which is to unload Na^+^ from xylem vessels into the xylem parenchyma cells of roots, limit Na^+^ accumulation in shoots, and protect plants against damage to photosynthetic cells. By contrast, class II HKT transporters exist in dicotyledonous species, and transport both Na^+^ and K^+^ [12].

*Populus* is an economically and ecologically important perennial woody plant that is widely cultivated and valued for its fast growth and high yield. However, soil salinization is an increasingly serious threat that limits poplar growth, so breeding salt-tolerant poplar has become necessary. The application of genetic engineering and transformation technology provide effective means of improving salt tolerance of poplar [13,14]. Many salt stress-related genes in poplar have been reported, including *WRKY40* [15], *ERF76* [16], *JERF* [17], *SOS2* [18], *TaLEA* [19], *GSK1* [20], and *mtlD* [21]. However, there is no report on the identification and functional characterization of HKT transporters in poplar.

Here, we isolated and characterized the *PeHKT1;1* gene from hybrid poplar. The expression patterns of *PeHKT1;1* were examined in different tissues under a range of salt stress conditions. To further understand the function of *PeHKT1;1* in poplar, we overexpressed *PeHKT1;1* in *Populus davidiana* × *Populus bolleana* using an *Agrobacterium*-mediated leaf disk transformation method, and then determined transgenic poplar lines and assessed phenotypic characteristics under salt stress. Moreover, three-year-old non-transgenic (NT) and transgenic poplars were assayed for catalase (CAT), superoxide dismutase (SOD), and peroxidase (POD) activities. The overexpression of *PeHKT1;1* dramatically improved salt tolerance in transgenic compared with NT poplar. These studies provide insight into the roles of *PeHKT1;1* in response to salt stress in *Populus*.

## 2. Materials and Methods 

### 2.1. Plant Material and Stress Treatments

Hybrid poplar (*Populus deltoides* × *Populus euramericana* cv. “Nanlin895”) plants were cultivated on Murashige and Skoog (MS) medium (pH 5.8) supplemented with 0.2% (*w*/*v*) gelrite and 3.0% (*w*/*v*) sucrose in a humid chamber at a temperature of 25/18 °C (day/night), daily photoperiod of 16/8 h (light/dark), and relative humidity of 60–80%. The hybrid poplar, *Populus davidiana* × *Populus bolleana*, with a stable and efficient genetic transformation system [22], was used for the NT controls and the transgenic plants in this study. The plants were cultivated as described above.

In order to characterize the tissue-specific expression of *PeHKT1;1* gene in “Nanlin895” poplar, various tissues (roots, stems, and leaves) were collected from 6-week-old plants. For the salt stress treatment, 6-week-old “Nanlin895” plants were transferred from initial solid MS medium (without NaCl) to liquid MS media containing either 0.6% or 1.8% *w*/*v* NaCl for different times: 0, 2, 6, 12, 24, 48, and 72 h. Two treatments of plants were used for growing, as previously described. After treatment, various tissues were harvested from three clonal plants of each treatment at each time point, frozen immediately in liquid nitrogen, and then stored at –80 °C for RNA isolation.

### 2.2. Extraction of DNA and RNA, and cDNA Synthesis

Poplar genomic DNA was extracted from young leaves using a DNeasy Plant Mini Kit (Qiagen, Hilden, Germany) following the manufacturer’s instructions. Total RNA was extracted from above harvested samples using a RNeasy Plant Mini Kit (Qiagen) and then treated with RNAse-free DNase I (Ambion, Austin, TX, USA). The quality and integrity of RNA samples were determined by a NanoDrop ND-1000 Spectrophotometer (Nanodrop, Wilmington, DE, USA) and 1% agarose gel electrophoresis. The first strand coding DNA (cDNA) was synthesized from 2 mg of total RNA using PrimeScript II Reverse Transcriptase (TaKaRa, Dalian, China), according to the manufacturer’s instructions.

### 2.3. Isolation of PeHKT1;1 and Sequence Analysis

The full-length *PeHKT1;1* cDNA was cloned from roots of “Nanlin895” by a 3′- and a 5′-Full RACE (Rapid Amplification of cDNA Ends) Kit (TaKaRa), according to the manufacturer’s protocols. The specific primers were designed based on sequences of the *Populus trichocarpa* genome (Potri.018G132200). The PCR products were amplified using PrimeSTAR Max DNA Polymerase (TaKaRa), followed by the A-addition procedure using Ex Taq Polymerase (TaKaRa). Each amplification product was gel-purified, cloned into the pMD-19T vector (TaKaRa), and subsequently sequenced. All primer sequences used in this study are listed in Table 1.

By comparing and aligning the 3′RACE, 5′RACE and middle-region sequences, the open reading frame (ORF) of *PeHKT1;1* was predicted using the ORFfinder program [23]. The predicted ORF cDNA and genomic sequences of *PeHKT1;1* were amplified by KOD-Plus-Neo DNA polymerase (TOYOBO, Osaka, Japan). The theoretical isoelectric point (pI), molecular weight (MW), amino acid composition and protein transmembrane structures of PeHKT1;1 were predicted and calculated using ExPASy ProtParam [24] and TMHMM [25]. The amino acid sequence alignments of PeHKT1;1 and other plant HKT proteins were aligned with ClustalX 2.1 software [26]. A neighbor-joining phylogenetic tree of HKT proteins was constructed using MEGA v.7.0 software [27] with 1000 bootstrap replicates.

### 2.4. Expression Analysis of PeHKT1;1

The expression patterns of *PeHKT1;1* were detected using semi-quantitative reverse transcription polymerase chain reaction (SqRT-PCR) and quantitative real-time polymerase chain reaction (qRT-PCR). The specific primers were designed to generate amplified fragments of 300–500 and 70–150 bp, respectively (Table 1). SqRT-PCR was performed by a non-saturating PCR reaction (28 cycles) with the *18SrRNA* (18S ribosomal RNA) gene as an internal control. qRT-PCR was performed on an ABI ViiA 7 Real-Time PCR system (Applied Biosystems, Carlsbad, CA, USA) using FastStart Universal SYBR Green Master with ROX for RT-PCR Kit (Roche, Indianapolis, IN, USA), according to the manufacturer’s protocol. The PCR procedure was 95 °C for 1 min, followed by 40 cycles at 95 °C for 15 s and 60 °C for 1 min. The specificity of the PCR reactions was confirmed by melting curve analysis of the amplicons. There were three biological replicates, and the relative expression levels of all samples were calculated using the 2^−ΔΔ*C*t^ method, with *Elongation Factor 1 alpha* (*EF1α*) as the reference gene [28].

### 2.5. Overexpression Vector Construction and Poplar Transformation

The ORF of *PeHKT1;1* cDNA was amplified by reverse transcription PCR (RT-PCR), and subsequently cloned into the pH35GS binary vector using the Gateway System (Invitrogen, Carlsbad, CA, USA) to replace the *ccdB* gene, which was located downstream of the CaMV35S promoter. The binary vector harboring Pro35S::*PeHKT1;1* was introgressed into *Agrobacterium tumefaciens* strain EHA105, which was used to transform poplar (Figure S1).

The *P. davidiana* × *P. bolleana* was transformed with the Pro35S::*PeHKT1;1* construct according to the established *Agrobacterium*-mediated transformation procedure [22]. In brief, leaves (0.5 cm × 0.5 cm blades) from 30-day-old sterile cultures were shaken with the *Agrobacterium* donor strain in liquid MS medium at 90 rpm for 30 min, and then transferred to solid MS medium without antibiotics. After 48 h in darkness, the leaves were washed three times with 200 mg L^−1^ cefotaxime solution and transferred to solid MS medium supplemented with 0.40 mg L^−1^ 6-benzyladenine (6-BA), 0.10 mg L^−1^ 1-naphthylacetic acid (NAA), 0.01 mg L^−1^ thidiazuron (l-phenyl-3-(1,2,3-thiadiazol-5-yl)urea), 200 mg L^−1^ cefotaxime, and 10 mg L^−1^ hygromycin. The regenerated shoots were individually removed from the callus and transferred to MS medium supplemented with 0.40 mg L^−1^ 6-BA, 0.10 mg L^−1^ NAA, 200 mg L^−1^ cefotaxime, and 10 mg L^−1^ hygromycin, and then regenerated with occurred roots in MS medium with 200 mg L^−1^ cefotaxime and 5 mg L^−1^ hygromycin.

### 2.6. Transgenic Poplar Confirmation and Salt Tolerance Assays

After screening using hygromycin resistance, the NT poplar and the putative transgenic poplar lines were validated by RT-PCR and qRT-PCR methods as described above, using the forward primer of the *35S* gene from the pH35GS binary vector and the reverse primer of the *PeHKT1;1* cDNA (Table 1). Salt tolerance tests were performed in hydroponic culture. Specifically, the 6-week-old NT and transgenic poplar lines were cultured for two weeks in liquid MS medium containing NaCl: 0, 0.2, 0.3, 0.4, and 0.5% *w*/*v*. The NT poplar served as the control. Each treatment was performed three replicates.

### 2.7. Physiological Assay

To examine the physiological parameters of NT poplar and *PeHKT1;1* transgenic lines, the three-year-old soil-grown poplar plants were treated with 0.8% (*w*/*v*) NaCl at different time points (0, 0.5, 1, 1.5, 2, and 6 h). The CAT, SOD, and POD activities were measured by leaves according to Li et al. [29] using the corresponding assay kits: total protein assay kit (BCA method, A045-2), catalase assay kit (visible light, A007-1), total superoxide dismutase (T-SOD) assay kit (hydroxylamine method, A001-1), and peroxidase assay kit (A084-3), according to the manufacturer’s respective manuals (Jiancheng Bioengineering Inc., Nanjing, China). The experiments were repeated at least five times. Statistical analyses were performed using SPSS 21.0 software (SPSS Inc., Chicago, IL, USA). Data were compared using one-way analysis of variance (ANOVA) followed by Duncan’s test.

### 2.8. Accession Numbers of HKTs from Different Species

The accession numbers of HKT genes were AtHKT1;1 (*Arabidopsis thaliana*, Q84TI7.1), MtHKT1;1 (*Medicago truncatula*, XP_013453240.1), PtHKT1;1 (*Populus trichocarpa*, EEF03794.1), SsHKT1;1 (*Suaeda salsa*, AAS20529.2), TcHKT1;1 (*Theobroma cacao*, EOY32095.1), SlHKT1;1 (*Solanum lycopersicum*, NP_001295273.1), SeHKT1;1 (*Salicornia europaea*, AKS12114.1), OsHKT1;1 (*Oryza sativa*, Q7XPF8.2), EcHKT1;1 and EcHKT1;2 (*Eucalyptus camaldulensis*, AF176035_1 and AF176036_1), TaHKT2;1 (*Triticum aestivum*, AAA52749), HvHKT2;1 (*Hordeum vulgare* subsp. *vulgare*, AEM44691.1) and PaHKT2;1 (*Phragmites australis*, BAE44385.1).

## 3. Results

### 3.1. Isolation and Characterization of PeHKT1;1

The full-length cDNA sequence of *PeHKT1;1* was successfully isolated and identified by RACE method, and had 2173 nucleotides, containing an ORF of 1608 bp that encoded a 62.0 kDa polypeptide of 535 putative amino acid residues and flanked by 332 bp of 5′-untranslated region (UTR) and 233 bp of 3′-UTR. The exon–intron structure of *PeHKT1;1* was determined by aligning cDNA and genomic sequences, which contained two introns (Supplementary Data 1).

**Figure 1:**
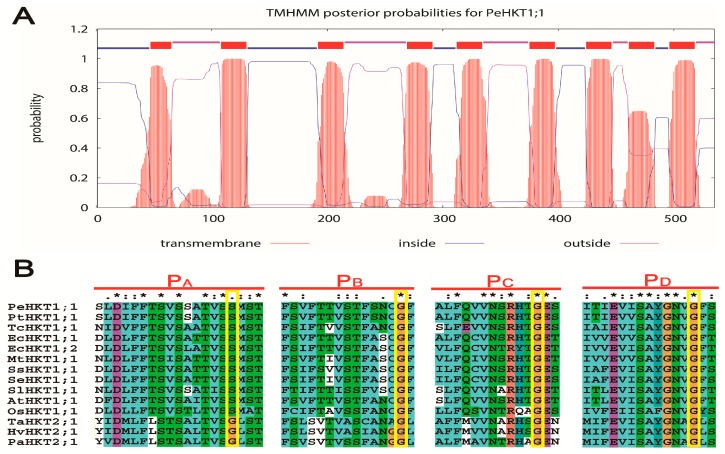

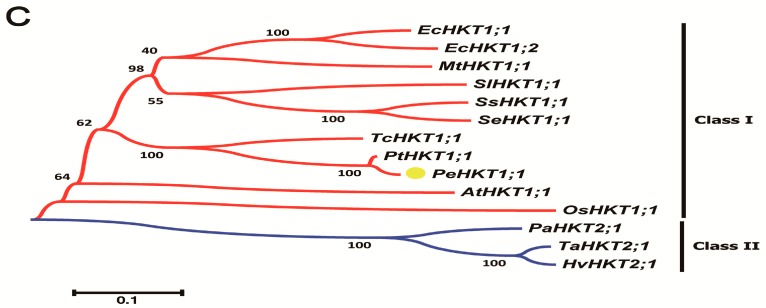
The transmembrane helices, alignment, and phylogenetic tree analysis of HKT (high-affinity K^+^ transporter) amino acid sequences. (**A**) The transmembrane helices of PeHKT1;1 protein were predicted by TMHMN Server v.2.0. (**B**) The four conserved selectivity-filter-pore regions of HKT were aligned, and highlighted in yellow, using ClustalX 2.1 software. A line above the alignment was used to mark strongly conserved positions. Three characters (“*”, “:” and “.”) were used: “*” indicates positions which have a single, fully conserved residue. “:” and “.” indicates positions that have ‘strong’ and ‘weaker’ conserved residues, respectively. (**C**) Phylogenetic tree analysis of 14 HKTs from 12 different plant species by neighbor-joining method using MEGA v7.0 software with 1000 iterations bootstraps. HKT: high-affinity K^+^ transporter.

To investigate the structure of PeHKT1;1 protein, the online software TMHMN Server v.2.0 was used to predict the transmembrane helices of PeHKT1;1 protein and indicated eight distinct transmembrane domains with a probability of one (Figure 1A). Moreover, the deduced amino acid sequences were aligned with sequences of other plant HKTs using ClustalX 2.1 software. The results revealed that PeHKT1;1 had four conserved selectivity-filter-pore regions (p-loops: P_A_ to P_D_, Figure 1B), and shared high homology with other plant HKT1 proteins, especially *A. thaliana* (69% identity), except for *P. trichocarpa* (98% identity) (Figure S2). Further phylogenetic analysis showed that PeHKT1;1, as typical for a dicotyledonous plant (Figure 1C), belonged to class I of HKT transporters, whose members are characterized by the presence of a serine (Ser) residue rather than a glycine (Gly) residue at the corresponding position in the P_A_-loop domain (Figure 1B).

### 3.2. Tissue-Specific Expression of PeHKT1;1

**Figure 2:**
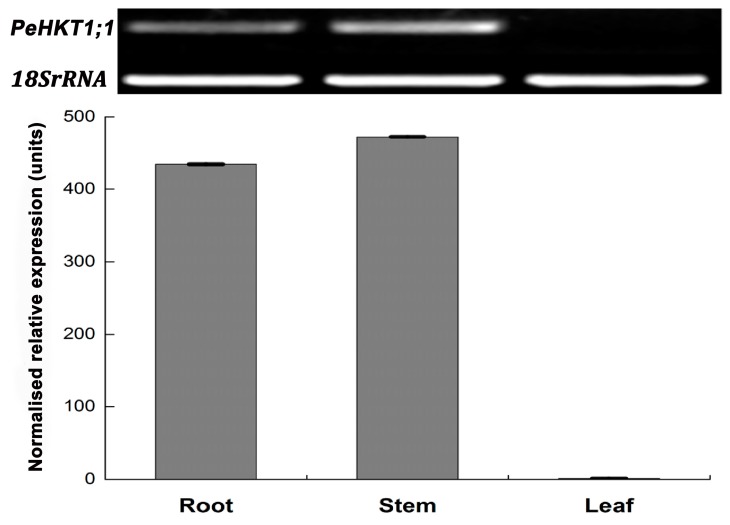
Tissue-specific expression levels of PeHKT1;1 analyzed by SqRT-PCR and qRT-PCR in “Nanlin895” poplar. The Y-axis represents relative quantitation and X-axis represents different tissues. The *18SrRNA* gene was used as an internal control for SqRT-PCR, the *EF1α* gene was used as a control for qRT-PCR, and the relative transcript levels were calculated using the 2^−ΔΔ*C*t^ method. Error bars showed standard deviations of three biological replicates.

The 6-week-old “Nanlin895” plants were used to study the tissue-specific expression profiles of *PeHKT1;1* under normal growth conditions. The mRNA level of *PeHKT1;1* was determined in roots, stems and leaves using SqRT-PCR and qRT-PCR. The two methods showed similar results—PeHKT1;1 was mainly expressed in stems and roots, but little detected in leaves in “Nanlin895” (Figure 2). These results implied that PeHKT1;1 plays an important role in *Populus* stems and roots.

### 3.3. PeHKT1;1 Transcripts in Salt Stress Conditions

**Figure 3:**
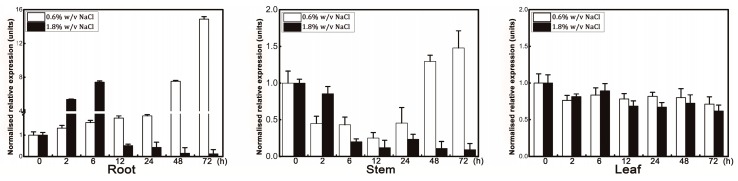
Expression level of *PeHKT1;1* in “Nanlin895” poplar under NaCl stress. Six-week-old poplar plants were treated with 0.6% and 1.8% *w*/*v* NaCl solution for 0, 2, 6, 12, 24, 48, and 72 h, and roots, stems, and leaves were harvested for qRT-PCR assay. Standard deviations were calculated from three independent qRT-PCR experiments.

Accumulated evidence from other plant species has demonstrated that HKT1 mediates Na^+^ uptake and transport [8], so we used qRT-PCR to investigate expression levels of *PeHKT1;1* in roots, stems, and leaves of “Nanlin895” subject to low (0.6% *w*/*v*) and high (1.8% *w*/*v*) NaCl stress at 0 (control), 2, 6, 12, 24, 48, and 72 h. For the 0.6% *w*/*v* NaCl treatment, the *PeHKT1;1* transcripts in roots were slightly enhanced within 24 h, and then upregulated dramatically at 48 and 72 h (approximately 8 and 15 times control values, respectively) (Figure 3). Interestingly, when poplar was exposed to 1.8% *w*/*v* NaCl stress, *PeHKT1;1* expression was induced significantly at 2 h, reached a maximum at 6 h, and then decreased as the treatment time progressed. In stems, *PeHKT1;1* was significantly downregulated, being reduced by around five times at 12 h compared with controls, and then gradually increased to original levels under 0.6% *w*/*v* NaCl stress. However, the mRNA level of *PeHKT1;1* showed substantial reduction, with a decrease of about five times after 12 h compared with controls for 1.8% *w*/*v* NaCl. Additionally, *PeHKT1;1* transcript levels showed no significant difference in leaves for 0.6% and 1.8% *w*/*v* NaCl stresses compared with controls. These results indicated that *PeHKT1;1* was involved in response to salt stress in *Populus*.

### 3.4. Generation of PeHKT1;1-Overexpressing Transgenic Poplar Lines

**Figure 4:**
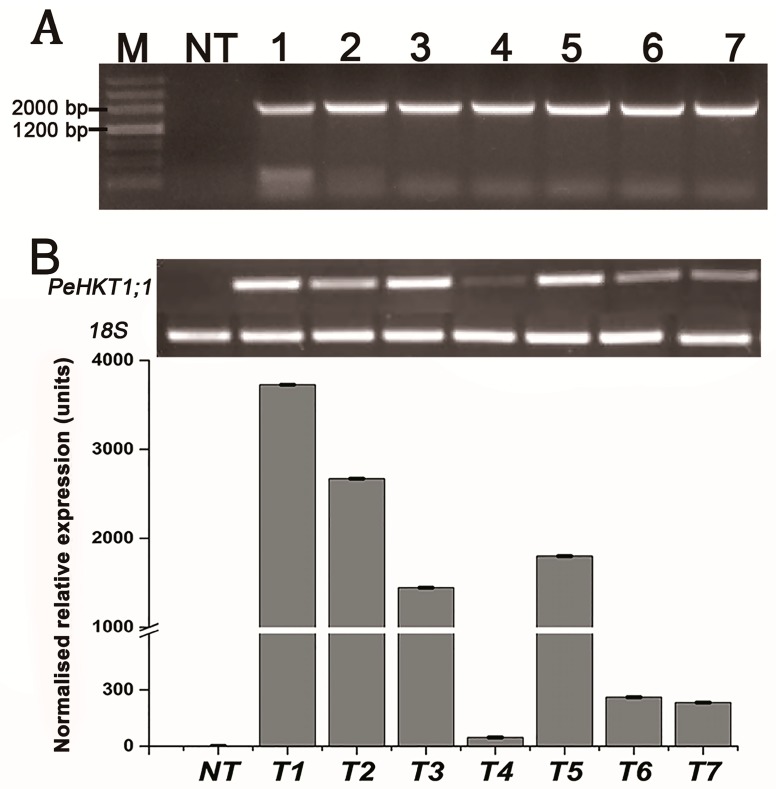
RT-PCR and qRT-PCR analyses of transgenic poplar lines. (**A**) Amplification of the inserted fragment from PeHKT1;1 and partial vector sequences by RT-PCR. (**B**) The expression level of PeHKT1;1 in the seven transgenic lines and NT poplar by SqRT-PCR and qRT-PCR. M: molecular marker; NT: non-transgenic poplar; 1–7: transgenic poplar lines.

To further investigate the potential functions of *PeHKT1;1* in *Populus*, we generated some *PeHKT1;1*-overexpressing transgenic lines of *P. davidiana* × *P. bolleana* by *Agrobacterium*-mediated leaf disk transformation, and seven independent transgenic lines were randomly selected for further testing. The sizes of PCR amplified genomic fragments from seven transgenic poplar lines were obtained by RT-PCR, which were confirmed with the expected sizes, containing the *PeHKT1;1* and partial vector sequences (Figure 4A). A control (NT plant) did not show any amplification band based on the transgene. The result implied that the Pro35S::*PeHKT1;1* vector was successfully integrated into the poplar genome. qRT-PCR further demonstrated that expression levels of *PeHKT1;1* were 48–3720 times higher than in NT poplar under normal conditions (Figure 4B). These results indicated successful integration and expression of *PeHKT1;1* in the seven transgenic poplar lines.

### 3.5. Overexpression of PeHKT1;1 Enhanced Salt Tolerance

**Figure 5:**
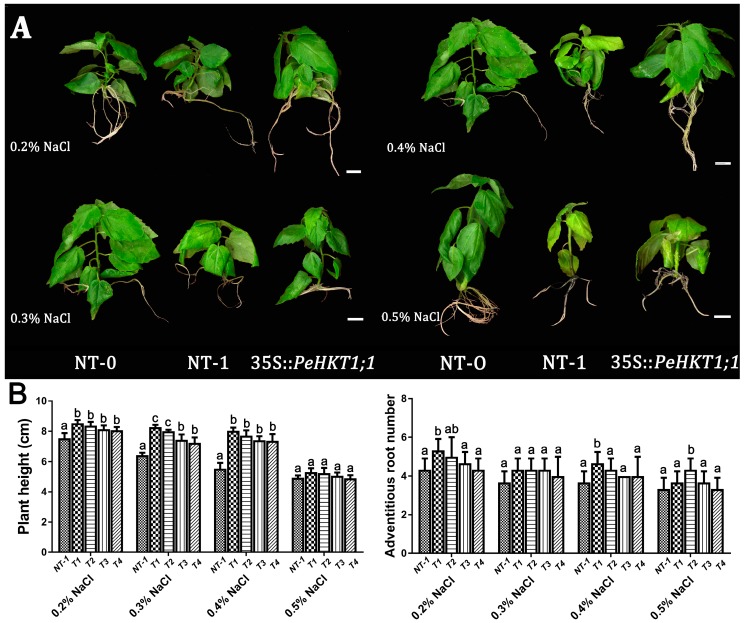
Phenotypes of NT poplar and PeHKT1;1 transgenic lines under salt stress conditions. Six-week-old non-transgenic (NT) and four transgenic poplar lines (T1, T2, T3, and T4) were treated with 0.2, 0.3, 0.4, and 0.5% *w*/*v* NaCl for two weeks. (**A**) Phenotypic observation of NT and transgenic poplar lines. The unstressed NT poplar (NT-0) were the positive control and NaCl-treated NT poplar (NT-1) was the negative control. All treatments had three biological replicates. Scale bar represents 1 cm. (**B**) The statistical analyses of plant height and adventitious root number. Data were analyzed using one-way ANOVA followed by Duncan’s test. Error bars with letters represent significant differences (*p* < 0.05, Duncan’s test).

In many plants, HKTs have been found to increase salt tolerance [30,31,32]. To detect whether HKT improves salt tolerance in poplar, we selected four transgenic poplar lines (T1, T2, T3, and T4) to determine their tolerance of different salinity stress conditions. The NT and *PeHKT1;1*-overexpressing poplar lines were grown with water (control), 0.2, 0.3, 0.4, and 0.5% *w*/*v* NaCl in liquid MS medium for two weeks. In addition, unstressed NT (NT-0) poplar was used as a positive control. The result showed the growth of NT-1 poplar was severely inhibited following the increase of Na^+^ concentration, showing varying degrees of dwarfing and etiolation (Figure 5A). Transgenic poplars showed a better relative growth rate, including plant height and adventitious root number (Figure 5B), which was consistent with the expression of *PeHKT1;1* in the previous study (Figure 4B). Nevertheless, the growth of NT and transgenic poplar lines were significantly inhibited when the Na^+^ concentration reached 0.5% *w*/*v*. These results indicated that *PeHKT1;1* overexpression improved salt tolerance in transgenic poplar. 

### 3.6. Overexpression of PeHKT1;1 Raised the Efficiency of Antioxidant Systems under Salt Tolerance

**Figure 6:**
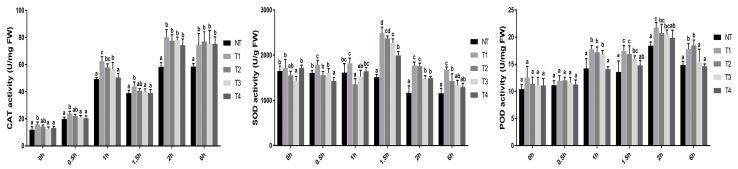
Catalase (CAT), superoxide dismutase (SOD), and peroxidase (POD) activities of four transgenic poplar lines (T1, T2, T3, and T4) and non-transgenic (NT) poplar. Three-year-old soil-grown poplar plants were subjected to 0.8% *w*/*v* NaCl at different time points (0, 0.5, 1, 1.5, 2, and 6 h). Data were analyzed using one-way ANOVA followed by Duncan’s test. Error bars with letters represent significant differences (*p* < 0.05, Duncan’s test).

The NT and transgenic poplar plants, which had been verified in the previous study, were transplanted into soil for three years to detect long-term effects. Subsequently, we randomly chose four independent transgenic lines to further investigate *PeHKT1;1* expression levels and physiological parameters. The Pro35S::*PeHKT1;1* vector had not been lost in the three-year-old transgenic lines, and *PeHKT1;1* was still overexpressed, confirmed by RT-PCR and qRT-PCR; however, expression levels were lower than the three previous years (Figure S4). To determine the antioxidant function of *PeHKT1;1* in poplar under salt stress conditions, activities of antioxidant enzymes, CAT, SOD, and POD, were examined in the three-year-old NT poplar and four transgenic poplar lines. Compared with NT poplars, transgenic poplars had higher CAT activity after one hour when plants were exposed to salt stress (Figure 6). Similarly, the *PeHKT1;1* transgenic poplars showed higher SOD and POD activities after 1 and 1.5 h, respectively. Moreover, comparison of *PeHKT1;1* transcript with CAT, SOD, and POD activities suggested that the transgenic poplar lines with higher *PeHKT1;1* expression had higher antioxidant enzymes activities. These results indicated that *PeHKT1;1* transgenic poplar had enhanced efficiency of antioxidant systems and higher salt tolerance than NT plants under salt stress.

## 4. Discussion

The HKTs are an essential gene family for salt tolerance in plants, and are mainly responsible for ion homeostasis and Na^+^ distribution within the plant [33,34,35]. Increasing numbers of *HKT* genes from various species have been reported to be salt inducible, and to improve salinity tolerance in transgenic plants. However, the functions of HKT1 transporters have not been described thoroughly in poplar. Therefore, we cloned the full-length cDNA of *PeHKT1;1* from hybrid poplar, and further characterized its roles in *Populus*.

The HKT proteins contain four membrane–pore–membrane motifs, each consisting of a P-loop between two transmembrane domains (M1 and M2), which are associated with the K^+^ channel [36,37]. Further research has demonstrated that the HKT1 transporters are Na^+^-specific transporters and have a S–G–G–G signature, whereas HKT2 transporters are Na^+^–K^+^ co-transporters and have a G–G–G–G type, due to a Gly residue that is crucial for K^+^ selectivity in the first P-loop domain [38,39]. In this study, we applied TMHMN to predict the transmembrane helices of PeHKT1;1 protein, which showed that it contained eight distinct transmembrane domains (Figure 1A), consistent with the previous reports. Moreover, the sequence alignment and phylogenetic analysis suggested that PeHKT1;1 protein had a S-G-G-G signature for the P-loop domains and belonged to class I of HKT transporters (Figure 1) [11].

So far, the tissue specificity of HKT1 transcripts is diverse in various plant species. The *AtHKT1;1* from *Arabidopsis* is mainly expressed in the root stele and leaf vasculature [40]. In rice, five *OsHKT1* genes were identified as belonging to class I, and showed no obvious patterns in mRNA levels among them [12]. *McHKT1;1* expression is most abundant in the leaves, and is also present in stems but absent from roots [41], and *SsHKT1;1* is predominantly expressed in leaves [42]. Moreover, *EcHKT1;1* and *EcHKT1;2* from *Eucalyptus camaldulensis* have higher expression in stems and leaves than roots [43]. The six *GmHKT*s in soybean showed low expression levels in leaves [32]. The tissue-specific expression study of *PeHKT1;1* suggested that the potential function of this gene is in roots and stems in poplar.

Regulatory mechanisms of HKT1 transporters have been identified in multiple species, and they play an important role in Na^+^ transport for increasing salt tolerance in plants [44]. The expression levels of *PeHKT1;1*, studied here, could aid in further understanding its function in poplar under salt stress. Our NaCl treatment resulted in increased *PeHKT1;1* expression in roots and decreased expression in stems, inferring that PeHKT1;1 may function in Na^+^ loading into the root xylem and limiting Na^+^ accumulation in stems [45]. Comparing *PeHKT1;1* expression for 0.6% and 1.8% *w*/*v* NaCl treatments showed that expression was more rapidly induced under high than low salt stress, inferring that transferring of plants from solution without NaCl to those with high NaCl concentration in a single step will definitely cause salt shock as a result of cell plasmolysis [46,47]. Altogether, these results implied that the function of *PeHKT1;1* may be essential in response to salt stress in poplar.

Many reports have shown that overexpression of salt stress-related genes can enhance salt stress tolerance in plants. However, few studies have been carried out on trees [19,48,49]. Due to many differences between herbaceous plants and trees, including growth, structure, and physiology in response to salt stress, we constructed an overexpressing *PeHKT1;1* vector under control of the CaMV35S promoter (Pro35S::*PeHKT1;1*), and transformed it into the poplar genome for further understanding of the roles of *PeHKT1;1* in trees. Although the expression level of endogenous *PeHKT1;1* significantly increased in “Nanlin895”, the native allele expression and the CaMV35S-driven allele of *PeHKT1;1* both contributed to high expression of *PeHKT1;1* in transgenic poplar. Compared with NT, transgenic poplar showed no dwarfing under normal growth conditions (Figure S3), which is inconsistent with the CaMV35S promoter possibly causing dwarfing of transgenic poplar [21,50]. The possible reason is the random insertion of different genes, and differences in expression levels may lead to different phenotypic characteristics.

Recent reports indicated that poplar growth is significantly reduced at 200 mM (1.2% *w*/*v*) NaCl treatment [19]—this was in accordance with our study, which showed comparable growth reduction under salt stress. Under our experimental conditions, overexpression of *PeHKT1;1* in transgenic poplar resulted in a better relative growth rate than in NT poplar, including plant height and roots, and this growth rate was consistent with the expression level of *PeHKT1;1* (Figure 4B and Figure 5B). Moreover, leaves of transgenic poplar showed no chlorosis, implying that leaf chlorophyll concentration remained higher than in NT poplar. These results suggested that reductions in plant growth may be associated with decreases in photosynthetic activities under salt stress. Of course, the promotion of salt tolerance has its limits—when the Na^+^ concentration exceeded 0.5% *w*/*v*, symptoms of salt damage still occurred in transgenic poplar. Thus, we conclude that overexpression of *PeHKT1;1* in transgenic poplar enhanced salt tolerance.

To explore the practical application of transgenic poplar overexpressing *PeHKT1;1*, the 6-week-old transgenic plantlets were transplanted into soil containing 0.2% *w*/*v* NaCl (Figure S5). At their early development stages (approximately 45 days), the growth rates of transgenic poplar showed dramatically decreased salt stress damage and increased plant height, compared with NT poplar. These results are consistent with a previous report [16]. Interestingly, salt tolerance of transgenic poplar lines showed the same trends with mRNA levels of *PeHKT1;1* as the overexpression of *TaLEA* gene in improving salt tolerance in transgenic poplar [19]. However, our results lacked high levels of replication, and this needs some future investigation.

During salt stress, the rapid generation and accumulation of ROS causes secondary oxidative stress and damage to nucleic acids, proteins, and other parts of cells in plants [2]. To scavenge ROS, plants have evolved multiple antioxidant defense systems, including various enzymes such as SOD, POD, and CAT [51]. Previous studies reported that overexpression of *PtSOS2* and *RtWRKY1* showed a significant activation of SOD, POD, and CAT in poplar and *Arabidopsis*, respectively [18,52]. In this study, transgenic poplar lines with higher *PeHKT1;1* expression exhibited higher CAT, SOD, and POD activities than NT poplar under salt stress (Figure 6). Thus, we speculated that the high expression of *PeHKT1;1* promoted the expression of ROS scavenging-related genes, and then increased the activities of antioxidant enzymes and decreased the damage from salt stress to poplar.

Salt tolerance is determined by coordinated expression of multiple genes in plants. Recent studies demonstrated that *HKT1* expression is restricted by some factors. Cytokinin application is involved in the repression of *AtHKT1;1* in roots by both type-B response regulators, ARR1 and ARR2 [53,54]. *ABI4* and *AtZIP24*, as negative regulators, also repressed *AtHKT1;1* expression [55,56]. Moreover, an MYB-type transcription factor, *OsMYBc*, binds to the *OsHKT1;1* promoter and regulates its expression [57]. Therefore, to further improve salt tolerance of our transgenic poplar lines will require research to explore more regulatory factors affecting HKT1 transporters and other salt stress-related genes in poplar.

## 5. Conclusions

In this study, we isolated and cloned the *PeHKT1;1* gene from hybrid poplar. The PeHKT1;1 protein contained four conserved selectivity-filter-pore (p-loop) domains and belonged to class I of HKT transporters, according to the amino acid sequence alignment and phylogenetic analysis. Temporal and spatial expression analysis showed that *PeHKT1;1* was highly induced in poplar root and stem under salt stress. Overexpression of *PeHKT1;1* in transgenic poplar resulted in better growth rates than control plants under salt stress. In accordance, assays showed stronger physiological activities of transgenic than NT poplar. Overall, we clearly demonstrated that transgenic poplar lines overexpressing *PeHKT1;1* were superior to NT poplar under salt stress treatment. All evidence indicated that *PeHKT1;1* plays an important role in salt tolerance in *Populus*.

## Figures and Tables

**Table 1:** Primers used in this study.

Primer ID	Forward (5′-3′)	Reverse (5′-3′)
3′RACE outer	ATCTATTGTCGATCTCTCCATC	TACCGTCGTTCCACTAGTGATTT
3′RACE inner	TGCCGAAAAAGCAACAGGAAGAGGTTGA	CGCGGATCCTCCACTAGTGATTTCACTATAGG
5′RACE outer	AGAACCCGACATTTCCGTATGC	CATGGCTACATGCTGACAGCCTA
5′RACE inner	GGTGAGAACAACAGGCACTGAACCAAAGAC	CGCGGATCCACAGCCTACTGATGATCAGTCGATG
PeHKT1;1-ORF	ATGAAGAGCTTTGCTAGT	CTAGGATAGCTTCCAAGCTTTACCA
SqRT-PCR	TCTTCGGCAACAGTTTCAAG	CCACACAAGCAAGGCTCTTA
qRT-PCR	GGCTATAGCTGCAAACGACA	AAGCCTTCCGAAGAGCATTA
*Eflα*	GGCAAGGAGAAGGTACACAT	CAATCACACGCTTGTCAATA
*18SrRNA*	TCAACTTTCGATGGTAGGATAGTG	CCGTGTCAGGATTGGGTAATTT
BP detection	ATGAAGAGCTTTGCTAGT	TAATACGACTCACTATAGGG’
LR detection	CGCACAATCCCACTATCCTT	CTAGGATAGCTTCCAAGCTTTACCA
Transgene detection	CGCACAATCCCACTATCCTT	CTAGGATAGCTTCCAAGCTTTACCA

RACE: rapid amplification of cDNA ends; ORF: open reading frames; SqRT-PCR: semi-quantitative reverse transcription PCR; qRT-PCR: quantitative real-time PCR; BP: BP clonase; LR: LR clonase; 18SrRNA: 18S ribosomal RNA.

## Supplementary Materials

The following are available online at http://www.mdpi.com/2073-4425/9/10/475/s1, Figure S1: The pH35GS binary vector diagram, Figure S2: Multiple sequence alignment of 14 HKT proteins using ClustalX 2.1 software, Figure S3: Phenotypes of PeHKT1;1-overexpressing transgenic poplar and non-transgenic (NT) poplar under normal conditions, Figure S4: RT-PCR and qRT-PCR analyses of three-year-old transgenic poplar lines, Figure S5: Phenotypes of seven transgenic poplar lines and non-transgenic (NT) poplar transplanted into soil containing 0.2% *w*/*v* NaCl for 45 days in the greenhouse. Supplementary Data 1: The sequences of *PeHKT1;1*.
