# Supplementary material for: Overexpression of PeHKT1;1 Improves Salt Tolerance in Populus
Source: Genes (Basel). 2018 Sep 29;9(10):475. doi: 10.3390/genes9100475 (PMC6210203; doi:10.3390/genes9100475)
Supplement: Supplementary file 1 [file genes-09-00475-s001.zip › genes-350383-supplementary_data.docx]

Supplementary Data

The full-length cDNA of PeHKT1;1

GAAAGTAATTGAAGCTAAAAGAAATTTTTTGTTTAGAATCACCTTTCTATTCCGATACTGCATATATTCTGGTTTTGCTAGATCATTCTCTACACATACCTAACCTACGCTAAACAGTTGCCAAATACTATAAACAAGCATTCCTGCCCCTTTGATTTCTTTTTCTATTATTCCACTAAGCTTGCATGATTAGCAGCCACTCATTCACAGTGAATTCCAACTAAAGTTGGTGTTCTATCGACTAGCTAGCTAGCTCGATCAGTAGGGTCCGTATTGCTCTTTTATAAACACCATATCCGATTGAGGTGCAAATTAATTTCTTGTTAGTACAGATGAAGAGCTTTGCTAGTTTTGGTAAGAAATTAGAGCGCCCTCGTAATTTCTTTTGCAACAAAGTAAGTTGCTTCCATAAATCTTCCATGTATTCAATCCGGTCCTTCCTTCAAGTATTAGTCTTTCAAATACACCGTTTTTGGGTTCAACTTGCTTATTTTGTAATCCTTTCCTTGGTTGGACATACGGCTTTGAAGGTTTCAAAGCCAAGACCTGGCTCATTAAGGCCTGCAAGCCTTGACATCTTCTTTACTTCTGTTTCTTCGGCAACAGTTTCAAGCATGTCAACGGTGGAAATGGAGGTCTTCTCTAACACCCAACTTATCATCATGACCATTTTGATGTTATTGGGTGGAGAGGTCTTCACTTCCATCCTTGGACTTTATCTGTCAAGGTTCAAATTCTCTAAACATGAAACCAAGGAAAGTAGAGTCTCTTCTGTTTACCACAATCCTCCAAAACGTACCAACTTTCCAGGGCTAGAGATTGAGAAACCAACCAATGTAGACCTAGAATGTAATCTCAACTCTTTAGACAATGATCACAGTCTCAAGTTGAACTCACTTAAGAGCCTTGCTTGTGTGGCTATGGGCTATTTTTCAGTTGTTCACATAACTGGTTCTAGTTTAGTTGCTATGTATACAAGCCTTGTTCCTAGTGCAAGACAAGTCCTGGGCAGTAAAGGAATTAAGATTCAGACATTTTCTGTGTTCACCACAGTCTCTACCTTTTCCAACTGTGGTTTTGTGCCTACAAATGAGAACATGGTAGCTTTCAAGAGGAATCCAGGTCTCCTACTCATTCTCATCCCTCAAATTCTTCTTGGAAACACATTATACCCATCATGCCTGCGATTTCTGATCTGGATTTTGGAGAAAATCACAAGGAAAGTGGAGTTCAGATACATTCTGATGAATACTAGAGAGATGGGCTATGGCCATTTGCTATCCTTTTCTCATTCGTGCCTTCTCGCTATCACAGTCTCGGGGTTTATACTGGTGCAGTTCATACTCTTTTGCTCCATGGAGTGGAATTCAGGAGCTATGGATGGTCTGAATCCCTATCAGAAGTTGATGGGTGCATTGTTTCAGGTTGTAAATTCAAGGCATACCGGTGAATCTATTGTCGATCTCTCCATCATCTCTCCAGCAATCTTGGTGCTCTTCGTGGTTATGATGTATCTCCCACCATACACTTCATTGATGCCGAAAAAGCAACAGGAAGAGGTTGATTCAGAAACTGGCCAAAAATGCAAAGACCAAAGGAAGTCTTTGGTTCAGTGCCTGTTGTTCTCACCTTTATCTACTTTAGCCATCTTTGTGATTCTCATTTGCGTCTCAGAGGGAGAGAAGTTGAATAAAGACCCTCTCAACTTCAACGTACTCAATATCACCATAGAAGTCATAAGTGCATATGGAAATGTCGGGTTCTCAACTGGCTATAGCTGCAAACGACAACTTGAACCTGATAGCTCGTGCAAGGATGCATGGTTTGGGTTTGTTGGAAGGTGGAGTAGCCTGGGAAAAATTATCCTCATCTTAGTAATGCTCTTCGGAAGGCTTAAGAAATTCAGCATCAATGGTGGTAAAGCTTGGAAGCTATCCTAGCTAGTATCCAGTACGTAGCAGAGCTTTTAGAAAGTCCTTTTGAAGGCAATTAGCTAGTTTACATGAAGTGATTGGGCTTGGAATATATATAGTGATGTGATCTGAGCCCTAATTATATTGTATAAGCTATGAGTGAGCTTGCATAGTTTTGTGTGGACTGAAAATCTCAACTTGTAGATGGTCAACTATTTTATAAAGTAATGTTGGGTTGGTGGGTTATGGAAAAAAAAAAA

The ORF gDNA of PeHKT1;1

ATGAAGAGCTTTGCTAGTTTTGGTAAGAAATTAGAGCGCCCTCGTAATTTCTTTTGCAACAAAGTAAGTTGCTTCCATAAATCTTCCATGTATTCAATCCGGTCCTTCCTTCAAGTATTAGTCTTTCAAATACACCGTTTTTGGGTTCAACTTGCTTATTTTGTAATCCTTTCCTTGGTTGGACATATGGCTTTGAAGGTTTCAAAGCCAAGACCTGGCTCATTAAGGCCTGCAAGCCTTGACATCTTCTTTACTTCTGTTTCTTCGGCAACAGTTTCAAGCATGTCAACGGTGGAAATGGAGGTCTTCTCTAACACCCAACTTATCATCATGACCATTTTGATGTTATTGGGTGGAGAGGTCTTCACTTCCATCCTTGGACTTTATCTGTCAAGGTTCAAATTCTCTAAACATGAAACCAAGGAAAGTAGAGTCTCTTCTGTTTACCACAATCCTCCAAAACGTACCAACTTTCCAGGGCTAGAGATTGAGAAACCAACCAATGTAGACCTAGAATGTAATCTCAACTCTTTAGACAATGATCACAGTCTCAAGTTGAACTCACTTAAGAGCCTTGCTTGTGTGGCTATGGGCTATTTTTCAGTTGTTCACATAACTGGTTCTAGTTTAGTTGCTATGTATACAAGCCTTGTTCCTAGTGCAAGACAAGTCCTGGGCAGTAAAGGAATTAAGATTCAGACATTTTCTGTGTTCACCACAGTCTCTACCTTTTCCAACTGTGGTTTTGTGCCTACAAATGAGAACATGGTAGCTTTCAAGAAGAATCCAGGTCTCCTACTCATTCTCATCCCTCAAATTCTTCTTGGAAACACATTATACCCATCATGCCTGCGATTTCTGATCTGGATTTTGGAGAAAATCACAAGGAAAGTGGAGTTCAGATACATTCTGATGAATACTAGAGAGATGGGCTATGGCCATTTGCTATCCTTTTCTCATTCGTGCCTTCTCGCTATCACAGTCTCGGGGTTTATACTGGTGCAGTTCATACTCTTTTGCTCCATGGAGTGGAATTCAGGAGCTATGGATGGTCTGAATCCCTATCAGAAGTTGATGGGTGCATTGTTTCAGGTTGTAAATTCAAGGCATACCGGTGAATCTATTGTCGATCTCTCCATCATCTCTCCAGCAATCTTGGTGCTCTTCGTGGTTATGATGTATCTCCCACCATACACTTCATTGATGCCGAAAAAGCAACAGGAAGAGGTTGATTCAGAAACTGGCCAAAAATGCAAAGACCAAAGGAAGTCTTTGGTTCAGTGCCTGTTGTTCTCACCTTTATCTACTTTAGCCATCTTTGTGATTCTCATTTGCGTCTCAGAGGGAGAGAAGTTGAATAAAGACCCCCTCAACTTCAACGTACTCAATATCACCATAGAAGTCGTAAGGTATCATAAATTCTTATCACAGAGAACGACTGTCCAAATCATTTATTTTTATTATGTAATCAAAATCTATGATTGACTGCCTGCGTGCATAATTTATTTAACTAACCCCATATTGAAAAATCAATCATTTTGTAGTGCATACGGAAATGTCGGGTTCTCAACTGGCTATAGCTGCAAACGACAACTTGAACCTGATAGCTCGTGCAAGGATGCATGGTTTGGATTTGTTGGAAGGTGGAGTAACATGGGAAAATTTATCCTCATCTTAGTAATGTTCTTCGGAAGGCTTAAGAAATTCAGCATCAATGGTGGTAAAGCTTGGAAGCTATCCTAG

The amino acid sequences of PeHKT1;1

MKSFASFGKKLERPRNFFCNKVSCFHKSSMYSIRSFLQVLVFQIHRFWVQLAYFVILSLVGHTALKVSKPRPGSLRPASLDIFFTSVSSATVSSMSTVEMEVFSNTQLIIMTILMLLGGEVFTSILGLYLSRFKFSKHETKESRVSSVYHNPPKRTNFPGLEIEKPTNVDLECNLNSLDNDHSLKLNSLKSLACVAMGYFSVVHITGSSLVAMYTSLVPSARQVLGSKGIKIQTFSVFTTVSTFSNCGFVPTNENMVAFKRNPGLLLILIPQILLGNTLYPSCLRFLIWILEKITRKVEFRYILMNTREMGYGHLLSFSHSCLLAITVSGFILVQFILFCSMEWNSGAMDGLNPYQKLMGALFQVVNSRHTGESIVDLSIISPAILVLFVVMMYLPPYTSLMPKKQQEEVDSETGQKCKDQRKSLVQCLLFSPLSTLAIFVILICVSEGEKLNKDPLNFNVLNITIEVISAYGNVGFSTGYSCKRQLEPDSSCKDAWFGFVGRWSSLGKIILILVMLFGRLKKFSINGGKAWKLS
