# Supplementary material for: Overexpression of PeHKT1;1 Improves Salt Tolerance in Populus
Source: Genes (Basel). 2018 Sep 29;9(10):475. doi: 10.3390/genes9100475 (PMC6210203; doi:10.3390/genes9100475)
Supplement: Supplementary file 1 [file genes-09-00475-s001.zip › genes-350383-supplementary_figures.docx]

|  | 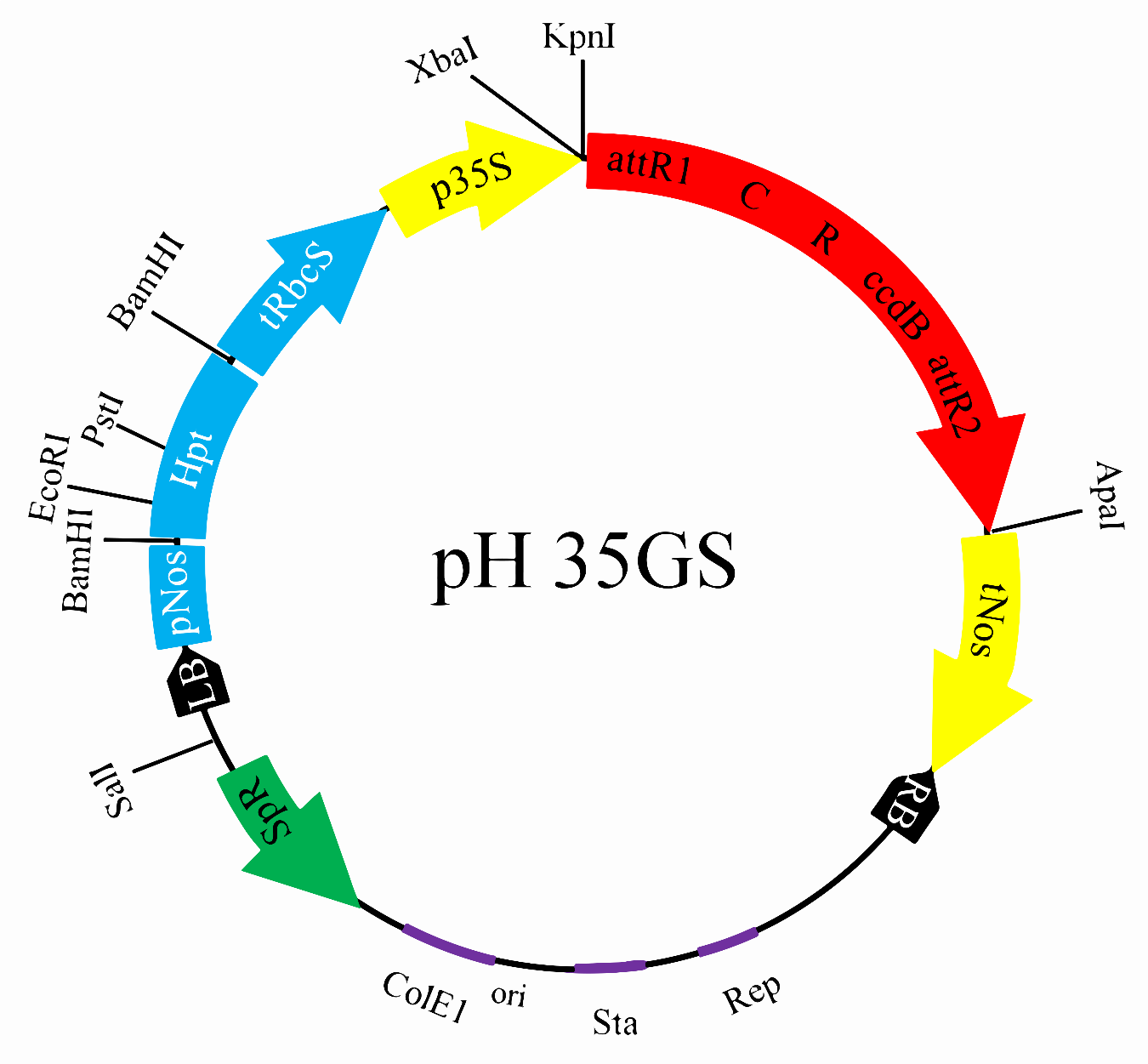 |
| --- | --- |

**Figure S1.** The pH35GS binary vector diagram.

| 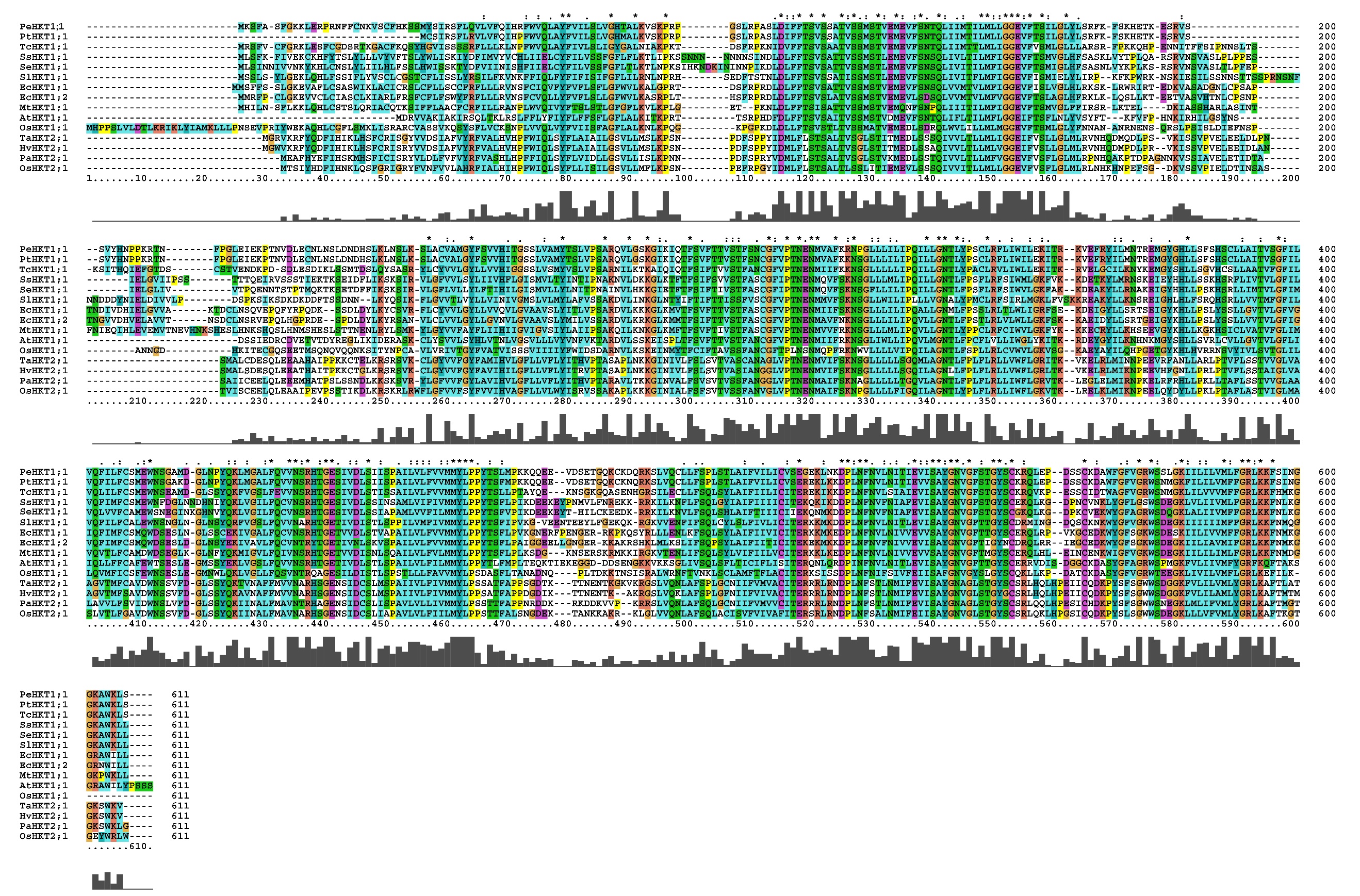 |  |
| --- | --- |

**Figure S2.** Multiple sequence alignment of 14 HKT proteins using ClustalX 2.1 software.

| 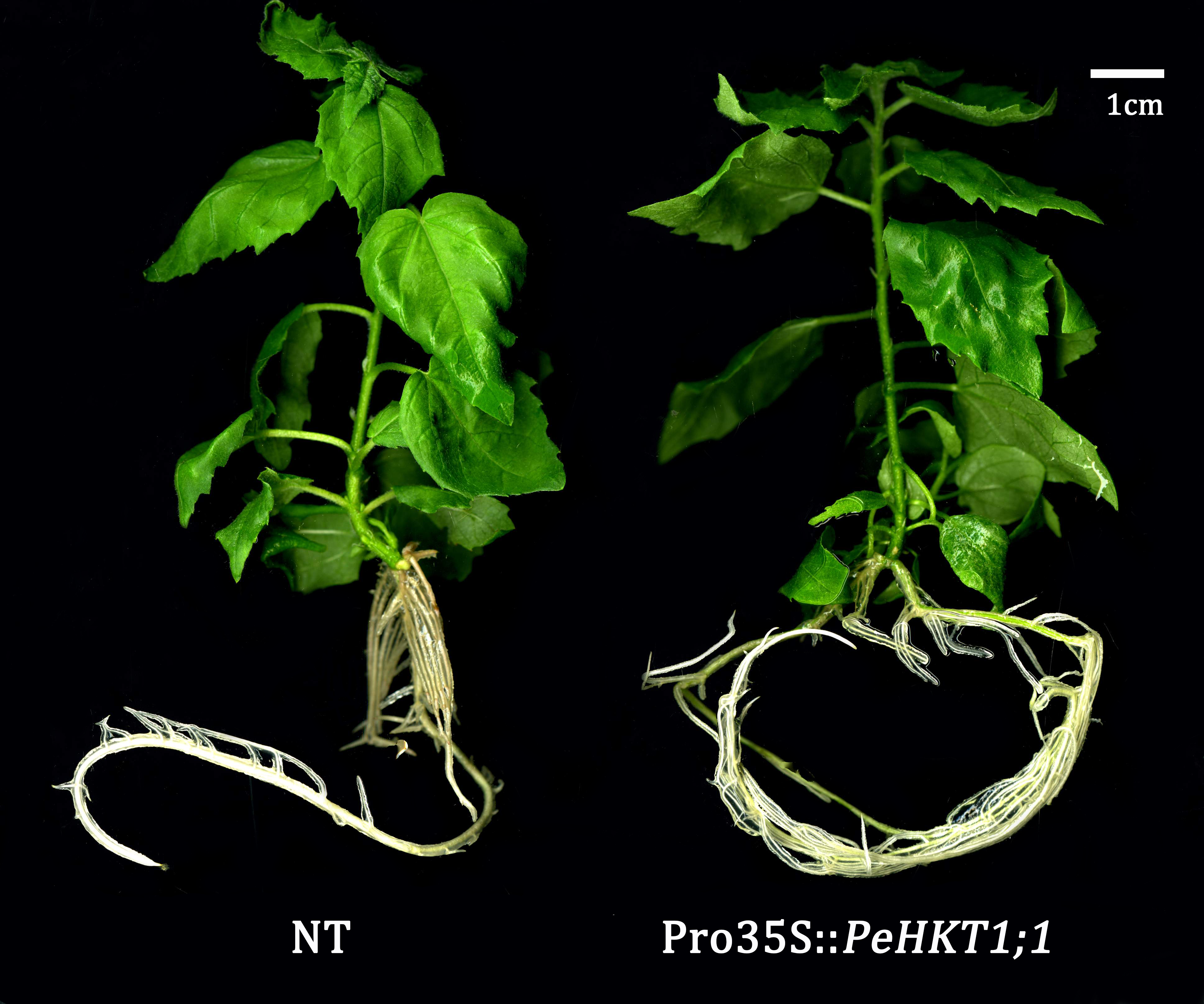 |  |
| --- | --- |

**Figure S3.** Phenotypes of PeHKT1;1-overexpressing transgenic poplar and non-transgenic (NT) poplar under normal conditions.

| 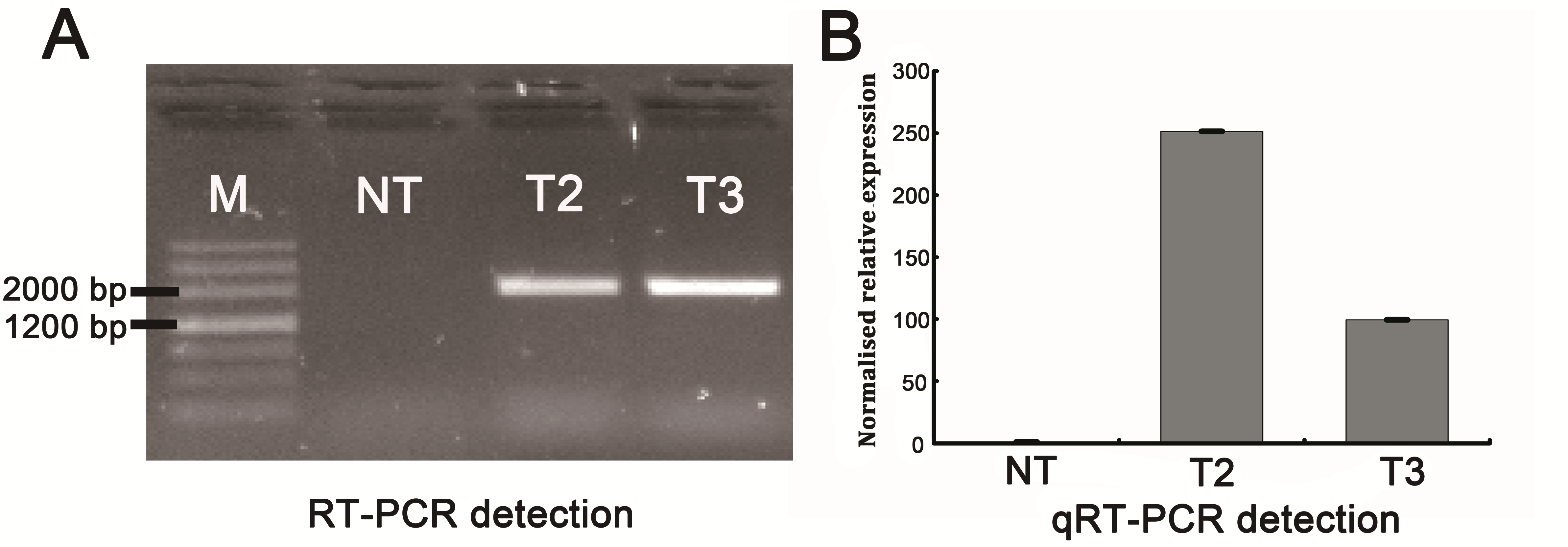 |  |
| --- | --- |

**Figure S4.** RT-PCR and qRT-PCR analyses of three-year-old transgenic poplar lines.

| 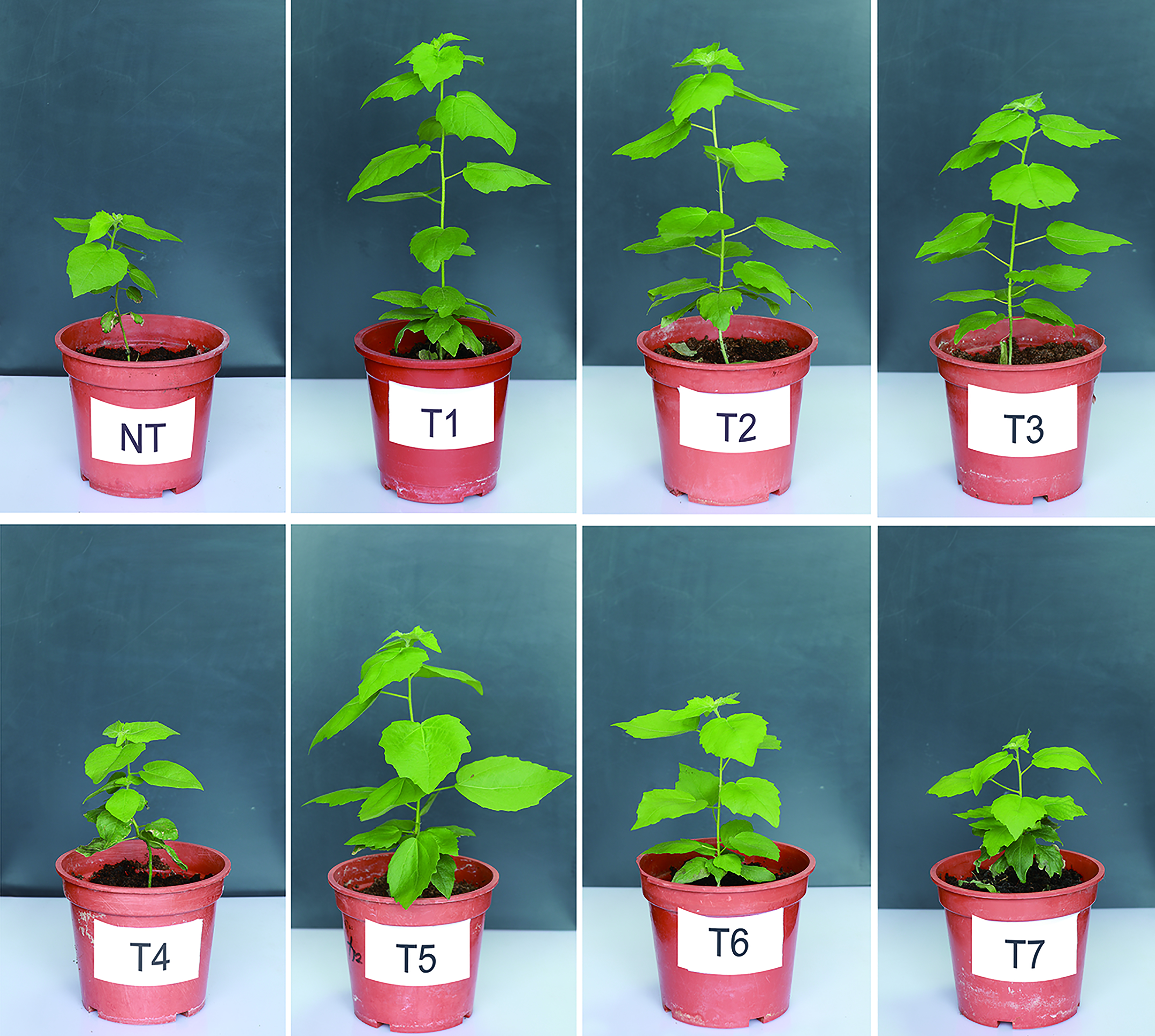 |  |
| --- | --- |

**Figure S5.** Phenotypes of seven transgenic poplar lines and non-transgenic (NT) poplar transplanted into soil containing 0.2% *w*/*v* NaCl for 45 days in the greenhouse.

© 2018 by the authors. Submitted for possible open access publication under the terms and conditions of the Creative Commons Attribution (CC BY) license (http://creativecommons.org/licenses/by/4.0/).
